# Machine learning for abdominal aortic calcification assessment from bone density machine-derived lateral spine images

**DOI:** 10.1016/j.ebiom.2023.104676

**Published:** 2023-07-11

**Authors:** Naeha Sharif, Syed Zulqarnain Gilani, David Suter, Siobhan Reid, Pawel Szulc, Douglas Kimelman, Barret A. Monchka, Mohammad Jafari Jozani, Jonathan M. Hodgson, Marc Sim, Kun Zhu, Nicholas C. Harvey, Douglas P. Kiel, Richard L. Prince, John T. Schousboe, William D. Leslie, Joshua R. Lewis

**Affiliations:** a Nutrition & Health Innovation Research Institute, Edith Cowan University, Perth, Australia; b Centre for AI&ML, School of Science, Edith Cowan University, Perth, Australia; c Department of Computer Science and Software Engineering, The University of Western Australia, Perth, Australia; d Department of Electrical and Computer Engineering, University of Manitoba, Winnipeg, Canada; e INSERM UMR 1033, University of Lyon, Hospices Civils de Lyon, Lyon, France; f Department of Radiology, Rady Faculty of Health Sciences, University of Manitoba, Winnipeg, Canada; g George and Fay Yee Centre for Healthcare Innovation, University of Manitoba, Winnipeg, Canada; h Medical School, The University of Western Australia, Perth, Australia; i Department of Endocrinology and Diabetes, Sir Charles Gairdner Hospital, Perth, Australia; j MRC Lifecourse Epidemiology Centre, University of Southampton, Southampton, United Kingdom; k NIHR Southampton Biomedical Research Centre, University of Southampton and University Hospital Southampton NHS Foundation Trust, Southampton, United Kingdom; l Hinda and Arthur Marcus Institute for Aging Research, Hebrew Senior Life, Department of Medicine, Beth Israel Deaconess Medical Center, Harvard Medical School, Boston, MA, USA; m Park Nicollet Clinic and HealthPartners Institute, HealthPartners, Minneapolis, USA; n Division of Health Policy and Management, University of Minnesota, Minneapolis, USA; o Departments of Medicine and Radiology, University of Manitoba, Winnipeg, Canada; p Centre for Kidney Research, Children's Hospital at Westmead School of Public Health, Sydney Medical School, the University of Sydney, Sydney, Australia; q Department of Statistics, University of Manitoba, Winnipeg, Canada

**Keywords:** Vascular calcification, Dual-energy X-ray absorptiometry, Machine learning, Aortovascular disease, Cardiovascular disease

## Abstract

**Background:**

Lateral spine images for vertebral fracture assessment can be easily obtained on modern bone density machines. Abdominal aortic calcification (AAC) can be scored on these images by trained imaging specialists to assess cardiovascular disease risk. However, this process is laborious and requires careful training.

**Methods:**

Training and testing of model performance of the convolutional neural network (CNN) algorithm for automated AAC-24 scoring utilised 5012 lateral spine images (2 manufacturers, 4 models of bone density machines), with trained imaging specialist AAC scores. Validation occurred in a registry-based cohort study of 8565 older men and women with images captured as part of routine clinical practice for fracture risk assessment. Cox proportional hazards models were used to estimate the association between machine-learning AAC (ML-AAC-24) scores with future incident Major Adverse Cardiovascular Events (MACE) that including death, hospitalised acute myocardial infarction or ischemic cerebrovascular disease ascertained from linked healthcare data.

**Findings:**

The average intraclass correlation coefficient between imaging specialist and ML-AAC-24 scores for 5012 images was 0.84 (95% CI 0.83, 0.84) with classification accuracy of 80% for established AAC groups. During a mean follow-up 4 years in the registry-based cohort, MACE outcomes were reported in 1177 people (13.7%). With increasing ML-AAC-24 scores there was an increasing proportion of people with MACE (low 7.9%, moderate 14.5%, high 21.2%), as well as individual MACE components (all p-trend <0.001). After multivariable adjustment, moderate and high ML-AAC-24 groups remained significantly associated with MACE (HR 1.54, 95% CI 1.31–1.80 & HR 2.06, 95% CI 1.75–2.42, respectively), compared to those with low ML-AAC-24.

**Interpretation:**

The ML-AAC-24 scores had substantial levels of agreement with trained imaging specialists, and was associated with a substantial gradient of risk for cardiovascular events in a real-world setting. This approach could be readily implemented into these clinical settings to improve identification of people at high CVD risk.

**Funding:**

The study was supported by a National Health and Medical Research Council of Australia Ideas grant and the Rady Innovation Fund, Rady Faculty of Health Sciences, University of Manitoba.

**Research in context**

**Evidence before this study**

We searched Google Scholar twice up to July 28, 2022, for literature published up to June 30, 2022, with no language restrictions, using the keywords “abdominal aortic calcification” and “vertebral fracture assessment”, and “machine learning” or “artificial intelligence”. The literature was scarce with only three published studies and a preprint (2020) retrieved. All studies utilised lateral spine imaging from a single bone density machine manufacturer, had 1300 or fewer labelled images for development, validation and testing, and all reported internal performances of the abdominal aortic calcification (AAC) algorithms without external validation.

**Added value of this study**

AAC scoring from bone density machine images is laborious and requires careful training. As a result, AAC scoring is not routinely performed when these images are acquired in clinical practice. Our study developed, validated and tested machine learning algorithms for AAC assessment and evaluated it in a real-world setting using a registry study of 8565 older men and women. We demonstrated greater ML-AAC-24 scores were associated with substantially higher cardiovascular disease risk and poorer long-term prognosis.

**Implications of all the available evidence**

This ML-AAC-24 scoring demonstrated promising performance, that may seamlessly add screening for cardiovascular disease risk in people undertaking bone density assessment where more costly, time-consuming or invasive tests may not be warranted.

## Introduction

Thoracolumbar lateral spine images can be easily obtained at the time of bone densitometry on modern dual-energy X-ray absorptiometry machines, with substantially lower radiation than standard radiographs. Increasingly, these images are obtained at the time of bone mineral density (BMD) testing for trained imaging specialists to identify clinically unrecognised vertebral fractures.^1^ These asymptomatic fractures are associated with high future fracture risk,^2^ appropriately increased utilisation of fracture prevention medications^3^ and can be used to develop a baseline so new vertebral fractures can be identified, with appropriate treatment initiated.^4^

In addition to the identification of vertebral fractures, abdominal aortic calcification (AAC) can be visually identified and semi-quantified using either an 8 (AAC-8) or a 24-point (AAC-24) system,^5^^,^^6^ by trained imaging specialists. This scoring is based on the linear length of calcified aortic wall relative to the height of the lumbar vertebrae.^7^^,^^8^ AAC-24 is the most widely used method, with the scores shown to be associated with atherosclerosis in other vascular beds,^9^ as well as higher future risk of coronary, cerebrovascular and cardiovascular disease (CVD) in older men and women.^7^^,^^10^ A recent meta-analysis of prospective studies, found in studies from the general population, the more extensive the AAC the higher the risk of future cardiovascular events and all-cause mortality.^11^ More recently AAC has been shown to be associated with higher healthcare costs in older men, greater long-term decline in muscle strength, increased falls and fracture risk people.^12^, ^13^, ^14^, ^15^, ^16^ These findings suggest AAC assessment may provide clinically important information on multiple chronic vascular and metabolic health conditions.

A major impediment to the use of AAC more widely has been the time-consuming nature of assessment, exacerbated by the lack of availability of trained readers. As such, recent efforts have focussed on whether machine learning (ML) may be applied to these images to identify and assess the extent of AAC. To date, these efforts have been limited to lateral spine images from single manufacturers, with relatively small datasets of available images annotated by trained imaging specialists.

We therefore sought to train and test ML algorithms for the automated assessment of AAC. We used a large collection of lateral spine images with AAC-24 scored by an imaging specialist, with over 15 years of experience reading these images. Moreover, we assessed the performance of our model on test image sets acquired from various generations of Hologic and GE machines, comprising the majority of DXA machines in clinical use. We then investigated whether these machine learning AAC-24 (ML-AAC-24) scores were associated with clinical cardiovascular outcomes in a real-world setting, where these images are captured as part of the routine clinical care.

## Methods

### Ethics statement

De-identified labelled (AAC-24 scores) and unlabelled images were sourced for the ML (Project number: 03349 LEWIS) from a number of existing studies collecting VFAs. For the Perth Longitudinal Study of Ageing Women, written informed consent was obtained from all participants for the study and follow up of electronic health records at baseline. The Human Ethics Committee of the University of Western Australia approved the study protocol and consent form (approval number 05/06/004/H50). The Human Research Ethics Committee of the Western Australian Department of Health also approved the data linkage study (approval number #2009/24). For other Hologic images the HealthPartners Institutional Review Board [IRB] [#A20-149]) approved the use of the de-identified Hologic images or the ethics approval for the study was granted by the Edith Cowan University Human Research Ethics Committee (Project number: 20513 HODGSON). For the GE images the study was approved by the Health Research Ethics Board for the University of Manitoba (HREB H2004:017L, HS20121). The Manitoba Health Information Privacy Committee approved access to the Manitoba data and waived the requirement for signed consent (HIPC 2016/2017–29). The authors acknowledge the Manitoba Centre for Health Policy for use of data contained in the Population Health Research Data Repository. The results and conclusions are those of the authors and no official endorsement by Manitoba Health and Seniors Care, or other data providers is intended or should be inferred.

#### AAC assessment

De-identified bone density machine-derived lateral spine images were available from four different models of bone density machine from the two largest bone density machine manufacturers: Hologic Inc (Bedford, MA, USA), and GE (GE Healthcare, Madison WI). Details of the datasets used are summarised in Supplementary Table S1 and Section 1. The current work was based on digitally enhanced lateral thoracolumbar spine images to assess the Kaupilla AAC-24 point semi-quantitative scoring method (AAC-24), as this is the most widely used method and has higher intra-rater reliability (Intra-class correlations coefficients [ICC] above 0.9) compared to AAC-8 scoring (ICC 0.8–0.9).^5^^,^^17^^,^^18^ All Hologic images were scored by J.T.S. whilst GE images were assessed by two readers trained by J.T.S. with J.T.S. as a referee for difficult or uncertain images. The intraclass correlation coefficient between raters in a random subset (10%) of the GE images was 0.9.^19^ Briefly, to calculate the AAC-24 point scores, the aorta is divided into 8 segments: four (4) on the anterior wall of aorta adjacent to L1, L2, L3, and L4, and four on the posterior wall. Each segment is assessed for the extent of calcification by judging the length of the calcification visible. A segment can receive a maximum score of 3 and a minimum of 0. If two-thirds or more of the aortic wall in a segment is calcified, it is scored as 3. If more than one-third and less than two-thirds of the wall is calcified, it is scored as 2. If one-third or less of the aortic wall is calcified, it is scored as 1. A score of 0 means that t no calcification is present.

#### EfficientNet for AAC quantification

The lateral spine images used in this work were pre-processed before being input into the machine learning model with only the AAC-24 scores used for training/validation/testing. Only single-energy (SE) images are available for the *Hologic* machines, whereas for *GE* machines both single- and dual-energy (DE) images were available. We have used a regression network to predict continuous outcome i.e., AAC score, given the image features. Supplementary Fig. S1 gives an overview of our framework, which comprises of i) image preprocessing, ii) feature extraction and iii) regression modules. For feature extraction, we use pre-trained EfficientNet-B3, which is one of the most efficient and high performing networks. We experimented with different versions of EfficientNets such as EfficientNet-B0, EfficientNet-B1, EfficientNet-B3 and EfficientNet-B4, and observed that for our datasets EfficientNet-B3 (12M parameters) achieved the best performance. Therefore, we chose EfficientNet-B3 as a backbone model for feature extraction in our framework, we refer the reader to the EfficientNet citation^20^ for further details of the network architecture. We replaced the last fully-connected convolutional layer of a pre-trained EfficientNet-B3 model, with a custom designed regression network. Our regression network predicts total AAC score on a continuous scale, ranging from 0 to 24, and is comprised of.

• two dense layers with Batch normalisation and Rectified Linear Unit (ReLU) activation function and,
• a fully connected final layer with linear activation

We optimised our network for Mean Squared Error loss, which is formalised as:

$$MSE=\frac{1}{n}\sum_{i\;=\;1}^{1}{\left(Y_{i}\;-\;Z_{i}\right)}^{2}$$

where, *n* is the total number of training images, *Y*_*i*_ and *Z*_*i*_ are the ML computed and ground truth scores, respectively. The ML computed scores are further categorised into three classes: low, moderate, and high using the following thresholding strategy:

$$f\left(Z_{i}\right)=0\left(low\right),\;if\;Z_{i}\;<\;2$$

$$f\left(Z_{i}\right)=1\left(moderate\right),\;if2\leq Z_{i}<6$$

$$f\left(Z_{i}\right)=2\left(high\right),\;if\;Z_{i}\geq 6$$

Our model regresses upon the ground truth AAC-24 scores, i.e., it tries to predict a continuous output value which is as close as possible to that of the given input image. Regression outcome may be preferred over a direct classification (only three possible outcomes) as the approach to map the input images to the ground truth scores (human assessed scores) makes it easier to measure the distance between the predicted values and ground truth. The continuous measure of error can be easily interpreted and compared across different models, making it a better choice for understanding the error of a score compared to classification. The pre-trained EfficientNet-B3 was fixed after pre-training and was not trained together with the regression module. To effectively analyse the reliability and generalisability of our approach, we first trained our model on the SE images from the Hologic-4500A dataset (n = 1914) and then independently tested it on the SE images from the Hologic Horizon (n = 508). The scans from GE have a GE SmartScan feature limiting the field of view to reduce radiation dose. This appears as a black mask on the regions surrounding the spine and aorta. To help our model adapt to GE scans, we fine-tuned it using a combination of 2331 iDXA and Lunar Prodigy scans from the Manitoba bone density registry. To account for the disbalance in the different AAC categories in the training dataset, we performed 10-fold stratified cross validation. 10-fold stratified cross validation involved splitting the sets of images into 10 random sets (folds) such that each set maintains the same distribution of AAC scores. Each time the model is trained on 9 folds and tested on the remaining (10th) one. This process is repeated 10 times and then the average performance of the model is reported. Details of various hyperparameters including, learning rate, batch size, cross-validation technique and the number of neurons in the fully connected layers of our regression network, are given in the Supplementary Text Section 2.

#### Studies with clinical outcomes

Clinical outcome data were available in two studies. The first was a prospective cohort study of Hologic 4500A lateral spine images where labelled images from 1998/1999 and 2003/2004 were available for training/testing and for comparison to imaging expert AAC scores for 15-year CVD and all-cause mortality outcomes. Unlabelled images from 2008 (not used for algorithm development) were available for 582 participants to determine the association with 5-year CVD and all-cause mortality outcomes. Cohort characteristics of women with AAC scores have been published previously.^9^^,^^10^ The second study was the Manitoba Bone Density Program database, a registry-based study in the Province of Manitoba, Canada. Since 2010, VFA images were included in the DXA assessment for qualifying individuals using the following criteria: T-score of ≤ −1.5 (minimum at the lumbar spine, total hip, or femoral neck) plus; a) age ≥70 years; b) age 50–69 years and historical height loss (recalled young adult height minus current height) > 5 cm, or measured height loss >2.5 cm, or glucocorticoid exposure for at least 3 months over the past year. All scans were performed with fan-beam DXA instruments (Lunar Prodigy or iDXA, GE Healthcare, Madison WI). Unlabelled lateral spine images were available from 8565 men and women (April 2010–March 2017). These images had no imaging expert scores and were not used for model development or evaluation. Manitoba cohort characteristics and covariates are provided in Supplementary Table S2 and Supplementary Text Section 3.

#### Cardiovascular outcomes

##### Perth longitudinal study of ageing women (PLSAW)

Mortality data were retrieved from the Western Australian Data Linkage system from January 1998 to December 31st 2013 as described previously.^21^ Cardiovascular mortality codes included International Classification of Diseases, Ninth Revision, Clinical Modification (ICD-9-CM) codes 390–459 until 1999 and then International Classification of Diseases, Tenth Revision, Australian Modification (ICD-10-AM) codes I00–I99 thereafter. The search for codes included all available diagnostic information that comprised Parts 1 and 2 of the death certificate. All diagnosis text fields from the death certificate were used to ascertain the cause(s) of deaths where these coded death data were not yet available.

##### Manitoba registry

The primary outcome was major adverse cardiovascular event (MACE) defined as all-cause mortality recorded in Vital Statistics, hospitalisation for acute myocardial infarction, or hospitalisation for non-hemorrhagic cerebrovascular disease occurring after VFA (index date). Secondary outcomes were incident hospitalisations for coronary artery disease, coronary revascularisation, congestive heart failure, or peripheral arterial disease, including a composite of any secondary endpoint. We also identified cardiovascular diagnoses that occurred prior to the index date using a look back to 1984. All diagnoses were based upon hospital discharge abstracts ([ICD-9-CM] prior to 2004 and International Classification of Diseases, Tenth Revision, Canadian Enhancements [ICD-10-CA] thereafter, diagnosis codes summarised in Supplementary Table S3). Outcomes were assessed from routine prospectively collected linked administrative data which does not require subject participation or response.

##### Statistical analysis

We evaluated our model on four test sets (4500A, Hologic Horizon, GE Prodigy and GE iDXA), by examining the Pearson correlation of the ML computed AAC scores (ML-AAC-24) with the ground truth (imaging specialist) assessment (AAC-24). When looking at the agreement by Cohen's weighted Kappa (linear) for AAC-24 scores we rounded these values to whole numbers to reflect the imaging specialist scoring and used the results from the dual-energy images for GE machines. We then assessed the performance of our model in terms of accurately classifying images into low, moderate, and high categories, based on their AAC scores. The AAC score thresholds for the three categories were defined as: i) Low (AAC-24 score<2), ii) Moderate (AAC-24 score≥2 to <6) and ii) High (AAC-24 score ≥6) based on previous publications.^7^^,^^9^^,^^10^ To measure agreement in the two or three group analysis we used Cohen's Kappa. Classification performance was assessed using metrics such as accuracy, sensitivity, specificity, Positive Predictive Value (PPV), Negative Predictive Value (NPV), and Intra-Class Correlation (ICC) (one-way random effects). In PLSAW age-adjusted Hazard ratios (HR) with 95% confidence intervals (CI) for sustaining a cardiovascular or any death during follow-up were estimated using Cox proportional hazards models. For the Manitoba registry, Cox models were also used to estimate HRs and 95% CI for sustaining MACE during follow-up. Observations were censored for migration out of province or end of follow-up (March 31, 2018). A partially adjusted model (age and sex) and a fully adjusted model (all covariates listed above) were used to estimate HRs for moderate vs. low predicted AAC severity and high vs. low predicted AAC severity. Additional models were tested in which individuals with prior cardiovascular diagnosis (myocardial infarction or cerebrovascular disease) were excluded. Similar analyses were performed examining the individual MACE endpoints and the secondary endpoints. Statistical analyses were performed with IBM SPSS for Windows (Version 27) and Stata software, version 14 (StataCorp LLC, College Station, Texas, USA). The proportional hazards assumption was confirmed from graphical analyses and the Schoenfeld residuals. For all analysis, p < 0.05 in two tailed testing was considered statistically significant.

### Role of funders

None of the funding agencies had any role in the conduct of the study; collection, management, analysis, or interpretation of the data; or preparation, review, or approval of the manuscript.

## Results

### Combined dataset of labelled images

**Fig. 1:**
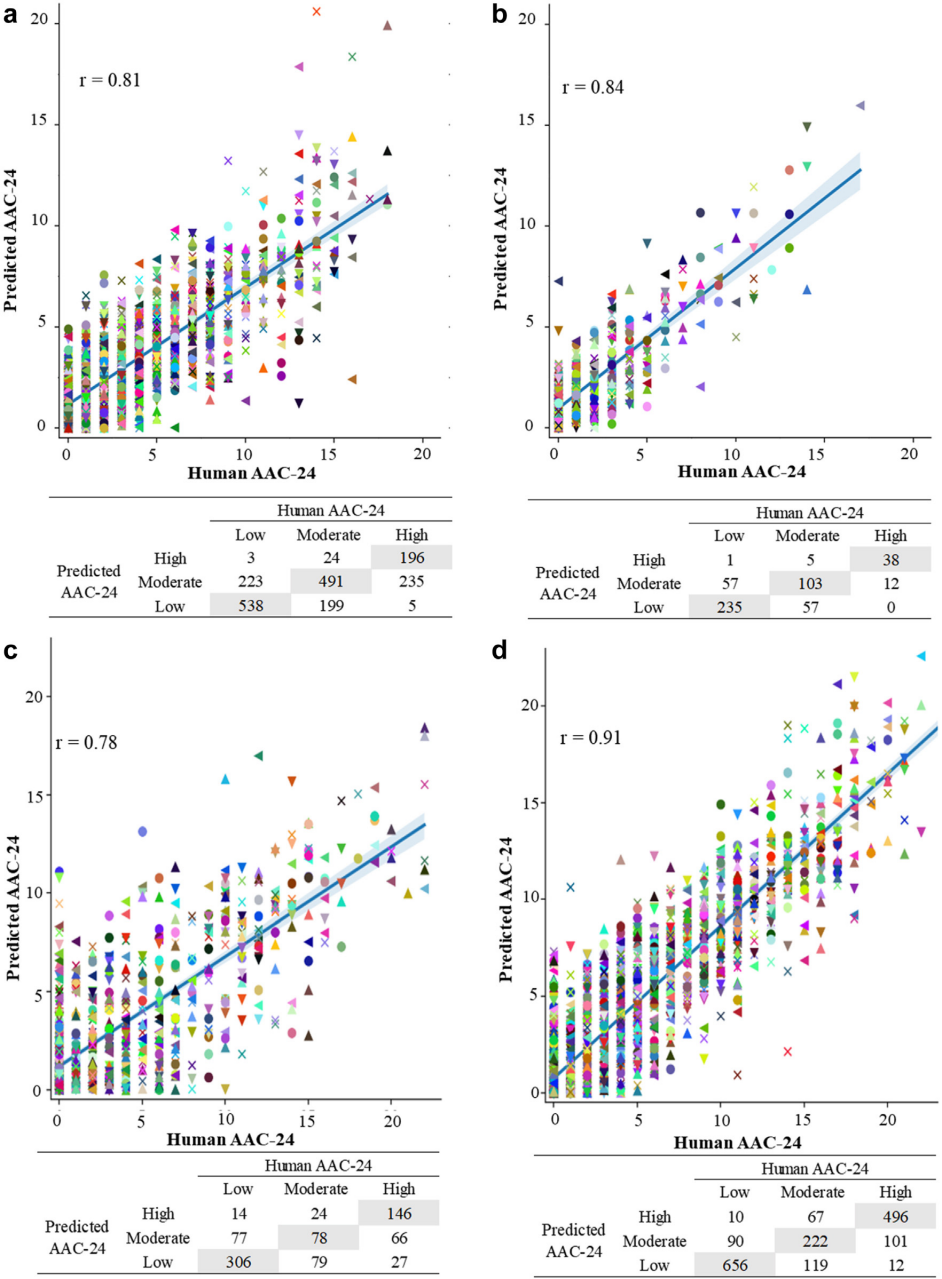
Scatter plots of imaging specialist assessments of AAC-24 scores (a) vs. the ML-AAC-24 scores for Hologic-4500A (training), (b) Hologic Horizon (testing), (c) GE Lunar Prodigy dual-energy (fine tuning & testing) and (d) GE iDXA dual-energy (fine tuning & testing). The blue line represents the regression line and ‘r’ is the Pearson correlation coefficient. Below each plot is the confusion matrix for three-class classification of the AAC scores. Each coloured shape represents an individual data point.

Fig. 1 presents the Pearson correlations (r) between the ground-truth (imaging specialists labelled AAC-24) and ML-AAC-24 scores across all test sets. In the combined dataset (n = 5012) the agreement (Cohen's weighted kappa) was substantial (0.61), intra-class correlation coefficient ICC (One-way random) between the ML and imaging specialist AAC-24 scores for all four test sets combined was 0.84 (95% CI [0.83, 0.84]), with a Pearson correlation of 0.86. When looking at the three established AAC groups (low, moderate, and high) the average classification accuracy was 80%, with two group performance provided in Supplementary Table S4.

### Hologic bone density machines

**Fig. 2:**
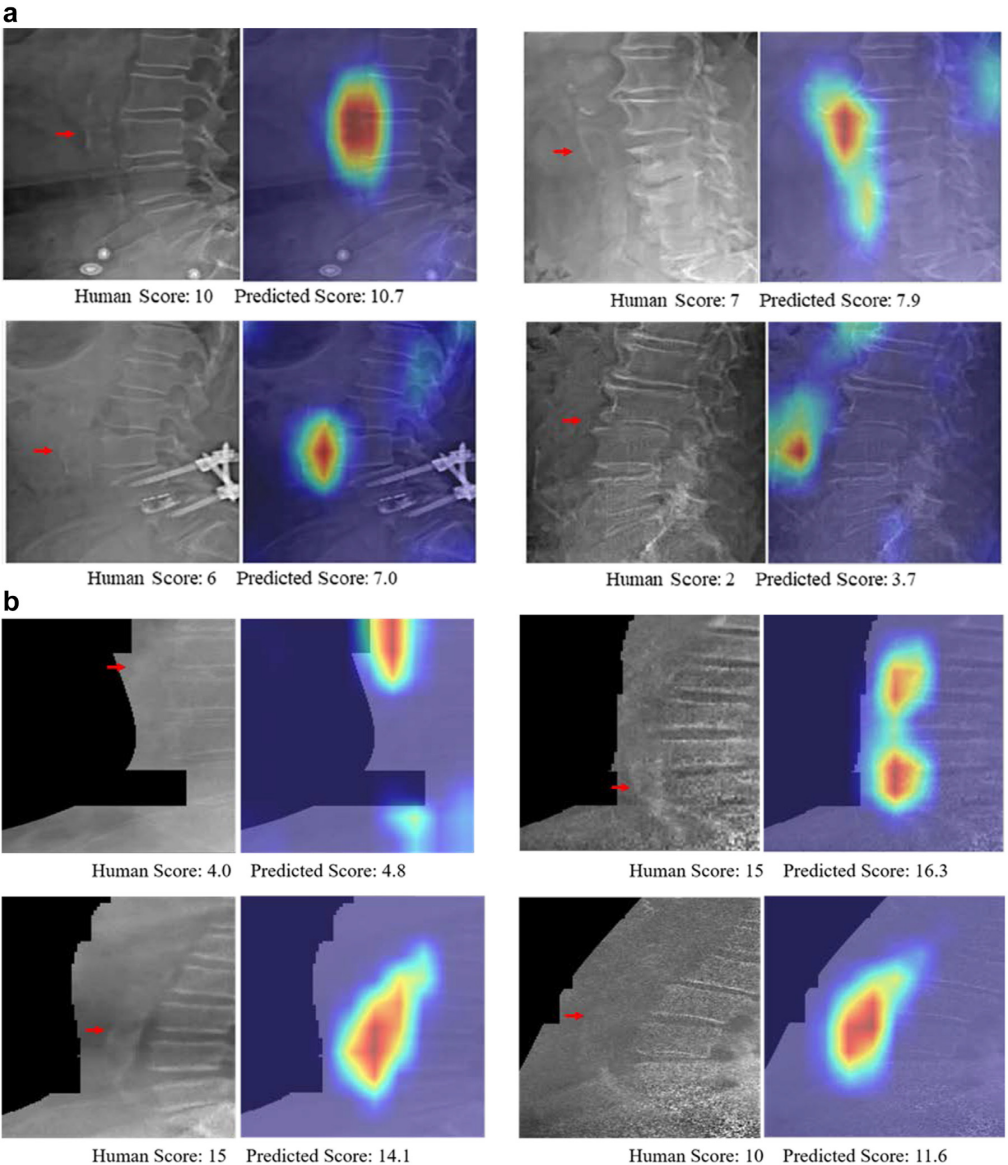
Qualitative performance of the model on side-by-side images without activation map (left) and with activation map (right) on (a) single-energy test images from Hologic Horizon test sets (top four pairs); (b) single and dual energy test images captured from Lunar Prodigy and iDXA machines (bottom four pairs).

The Cohens weighted Kappa for the Hologic-4500A and Horizon test scans were 0.51 and 0.56, respectively. The ICCs were 0.76 (95% CI [0.74, 0.78]) and 0.82 (95% CI [0.78, 0.84]), respectively. The trained model had an average classification accuracy of 76–83% for the established AAC groupings (low, moderate, and high) for the two Hologic test sets (Table 1, Fig. 2). The performance of the ML-AAC-24 model to identify established two group cut points (low vs. moderate-extensive and low-moderate vs. extensive) are presented in Supplementary Table S4.

**Table 1:** Test characteristics on Hologic and GE test sets.^a^

Hologic-4500A (SE) (n = 1914)	Low AAC (n = 764)	Moderate AAC (n = 714)	High AAC (n = 436)	Average
Accuracy (%)	77.5	64.4	86.1	76.0
Sensitivity (%)	70.4	68.8	45.0	61.4
Specificity (%)	82.3	61.8	98.2	80.8
Negative predictive value (%)	80.7	76.9	85.8	81.1
Positive predictive value (%)	72.5	51.7	87.9	70.7

Hologic horizon (SE) (n = 508)	Low AAC (n = 293)	Moderate AAC (n = 165)	High AAC (n = 50)	Average
Accuracy (%)	77.4	74.2	96.5	82.7
Sensitivity (%)	80.2	62.4	76.0	72.9
Specificity (%)	73.5	79.9	98.7	84.0
Negative predictive value (%)	73.1	81.5	97.4	84.0
Positive predictive value (%)	80.5	59.9	86.4	75.6

GE lunar prodigy (DE) (n = 817)	Low AAC (n = 397)	Moderate AAC (n = 181)	High AAC (n = 239)	Average
Accuracy (%)	75.9	69.9	84.0	76.6
Sensitivity (%)	77.1	43.1	61.1	60.4
Specificity (%)	74.8	77.5	93.4	81.9
Negative predictive value (%)	77.5	82.7	85.3	81.9
Positive predictive value (%)	74.3	35.3	79.3	63.0

GE iDXA (DE) (n = 1773)	Low AAC (n = 756)	Moderate AAC (n = 408)	High AAC (n = 609)	Average
Accuracy (%)	87.0	78.7	89.3	85.0
Sensitivity (%)	86.8	54.4	81.4	74.2
Specificity (%)	87.1	86.0	93.4	88.8
Negative predictive value (%)	89.9	86.3	90.6	88.9
Positive predictive value (%)	83.4	53.8	86.6	74.6

a The algorithm was trained on Hologic 4500A (SE), fine-tuned on GE Lunar Prodigy and iDXA and tested on all datasets. Abbreviations; AAC Abdominal Aortic Calcification, AAC-24 Abdominal Aortic Calcification 24-point scores, DE Dual-energy, SE Single-energy, GE General Electric.

### GE bone density machines

The linear correlations of the models which were independently fine-tuned on DE and SE images of the Manitoba registry, are shown in Fig. 1 (DE only) and Supplementary Fig. S2 (SE only). The model achieved a higher performance on iDXA SE and DE images compared to Lunar Prodigy. The Cohens weighted Kappa for the Prodigy and the iDXA were 0.50 and 0.70. The ICC between the ML and imaging specialist AAC scores on the iDXA test set was 0.90 (95% CI 0.89, 0.91) for both SE and DE. ICCs between the ML-AAC-24 and imaging specialist scores on the Lunar Prodigy test set were 0.78 (95% CI 0.75, 0.81) for DE and 0.73 (95% CI (0.69, 0.76) for SE. Additional quantitative scores of the models trained on DE scans are shown in Table 1, Fig. 2 and SE scans in Supplementary Table S5.

### Clinical outcomes–Perth longitudinal study of ageing women

To determine if ML-AAC-24 scores had a similar association with clinical events we tested the association between in ML-AAC-24 with 15-year CVD (n = 266) and all-cause mortality (n = 419) in 1082 women. Women in the high ML-AAC-24 group (vs. lowest) had a HR for CVD death of 2.17, 95% CI 1.49–3.15 vs. imaging specialist high AAC HR 1.66, 95% CI 1.21–2.29. Similar results were seen for all-cause mortality high ML-AAC-24 group 1.79, 95% CI 1.31–2.45 vs. expert assessed high AAC group HR 1.57, 95% CI 1.22–2.02. In 582 women (mean age 84.7 years ± 2.4 years) with images obtained in 2008 without no imaging specialist AAC-24 assessment women with high ML-AAC-24 score had similarly higher relative hazards of CVD mortality (aHR 2.24, 95% CI 1.22–4.14) and all-cause mortality (aHR 1.66, 95% CI 0.99–2.80) vs. those with a low ML-AAC-24 score.

### The Manitoba registry-based study

**Fig. 3:**
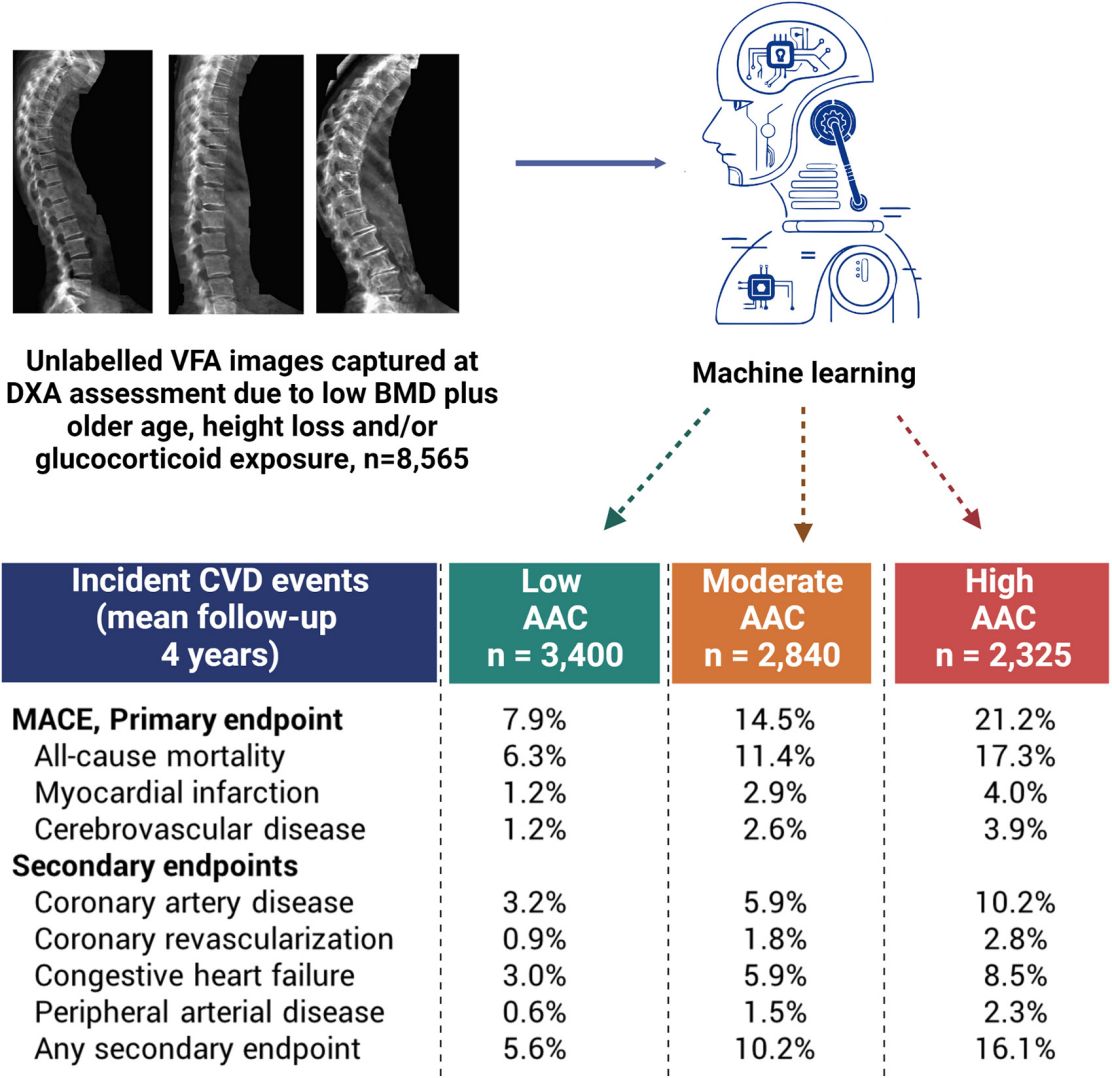
Proportion with clinical outcomes stratified by machine-learning abdominal aortic calcification groups from dual-energy Lunar Prodigy and iDXA images captured at the time of bone density testing. “Created with BioRender.com.”

Baseline characteristics for the outcomes assessment cohort are summarised in Supplementary Table S2. Increasing ML-AAC-24 score showed expected associations with increasing age, smoking, diabetes, previous cardiovascular diagnoses, hypertension diagnosis, and cardiac medications. During a mean follow-up 4 years, 1177 (13.8%) met the primary MACE outcome. The incidence of MACE was 7.9% in those with low ML-AAC, 14.5% in those with moderate ML-AAC-24 and 21.2% in those with high ML-AAC-24 (Fig. 3), corresponding to rates of 19.1, 37.9 and 61.7 per 1000 person-years, respectively. Similar trends were seen for the individual endpoints of death, myocardial infarction and cerebrovascular disease, as well as each of the secondary endpoints (all p for trend <0.001). Kaplan–Meier event-free survival for MACE, and its constituents (all-cause mortality, acute myocardial infarction and ischemic cerebrovascular events) showed separation in the curves that occurs from the outset of the observation with widening separation to the end of follow-up (Supplementary Fig. S3). Table 2 shows HRs for ML-AAC-24 score adjusted for age and sex, multiple covariates, and after excluding individuals with a prior cardiovascular diagnosis for the same condition.

**Table 2:** Hazard ratios (HRs, 95% CIs) for cardiovascular outcomes by machine learning abdominal aortic calcification 24 (ML-AAC-24) severity in the Manitoba registry-based cohort from duel-energy Lunar Prodigy and iDXA images.^a^

	Events	Model 1, Age and sex-adjusted			Model 2, Adjusted for multiple covariates^b^			Model 3, Adjusted for multiple covariates^b^ and excluding those with prior diagnosis for the same condition		
		Low	Moderate	High	Low	Moderate	High	Low	Moderate	High
MACE, Primary endpoint	1177	1 (Ref)	1.74 (1.49–2.03)	2.62 (2.25–3.06)	1 (Ref)	1.54 (1.31–1.80)	2.06 (1.75–2.42)	1 (Ref)	1.54 (1.30–1.83)	2.11 (1.77–2.51)
All-cause mortality	940	1 (Ref)	1.64 (1.37–1.94)	2.55 (2.15–3.03)	1 (Ref)	1.45 (1.21–1.73)	2.01 (1.68–2.41)	1 (Ref)	1.42 (1.17–1.72)	2.00 (1.64–2.43)
Myocardial infarction	216	1 (Ref)	2.41 (1.65–3.51)	3.73 (2.55–5.45)	1 (Ref)	2.05 (1.40–3.00)	2.79 (1.88–4.15)	1 (Ref)	2.23 (1.48–3.35)	3.24 (2.12–4.96)
Cerebrovascular disease	204	1 (Ref)	1.99 (1.35–2.94)	2.97 (2.02–4.38)	1 (Ref)	1.71 (1.15–2.54)	2.15 (1.44–3.21)	1 (Ref)	1.75 (1.15–2.66)	2.04 (1.32–3.15)
Secondary endpoints										
Coronary artery disease	514	1 (Ref)	1.79 (1.40–2.28)	3.19 (2.52–4.05)	1 (Ref)	1.35 (1.05–1.73)	1.98 (1.54–2.55)	1 (Ref)	1.30 (0.96–1.76)	2.21 (1.63–3.01)
Coronary revascularization	147	1 (Ref))	2.41 (1.53–3.80)	4.37 (2.78–6.85)	1 (Ref)	2.15 (1.35–3.41)	3.49 (2.17–5.62)	1 (Ref)	2.42 (1.48–3.96)	4.06 (2.44–6.78)
Congestive heart failure	467	1 (Ref)	1.65 (1.28–2.12)	2.32 (1.74–2.87)	1 (Ref)	1.32 (1.02–1.70)	1.57 (1.21–2.03)	1 (Ref)	1.51 (1.14–1.98)	1.62 (1.21–2.16)
Peripheral arterial disease	118	1 (Ref)	2.38 (1.41–4.01)	3.84 (2.29–6.44)	1 (Ref)	1.90 (1.12–3.22)	2.24 (1.30–3.87)	1 (Ref)	1.96 (1.12–2.43)	2.17 (1.20–3.93)
Any secondary endpoint	856	1 (Ref)	1.68 (1.39–2.02)	2.65 (2.21–3.18)	1 (Ref)	1.30 (1.08–1.57)	1.78 (1.47–2.15)	1 (Ref)	1.50 (1.19–1.89)	1.89 (1.49–2.40)

Bolded values represent significantly different HR compared to low AAC.

a Predicted ML-AAC-24, low <2, moderate 2–5, high ≥6.

b Covariates: age, sex, body mass index (BMI), current smoking, high alcohol intake, income, rural residence, ethnicity; diagnoses of diabetes, hypertension, prior myocardial infarction or cerebrovascular disease; medication use in the prior year (glucocorticoid, statin, nonselective beta blocker, selective beta blocker, angiotensin receptor blocker, ACE inhibiter, aldosterone blocker, loop diuretic, thiazide diuretic, digoxin, calcium channel blocker, long acting nitrate and oral anticoagulant).

## Discussion

Medical image analysis such as segmentation or localisation of lower lumber regions may be useful in accurately predicting AAC 24-point scores. However, such analysis is dependent on the availability of ground truth annotations for the lumber regions, which is very expensive to acquire at scale. The low resolution of VFA scans and presence of artefacts further adds to the complexity of localisation and identification of lumber regions and aortic wall. On the other hand machine learning models leverage data examples to learn the useful features to predict the overall AAC scores, without the need of other intermediate steps. In this study, we found good levels of agreement between imaging specialist and ML-AAC-24 scores across DXA machines from different manufacturers that have been widely used over the last three decades. We also observed high levels of accuracy (76–85%), with only 3% of manually-assessed individuals with high AAC-24 being incorrectly classified as low by the CNN. This is notable as these are the individuals with the greatest extent of disease and highest risk of fatal and non-fatal cardiovascular events and all-cause mortality.^11^ There were also few individuals (1%) who were manually classified as low being incorrectly classified as high AAC by the CNN. In addition to validating the developed ML model, we demonstrated that similar to imaging expert AAC-24 scores the ML-AAC was associated with mortality outcomes in one study, whilst in the real-world assessment without expert AAC-24 scores, the ML-AAC-24 was strongly associated with future CVD outcomes.

Whilst expert assessed AAC-24 is not routinely performed or reported on lateral spine images from bone density machines, it is being assessed and reported to patients in some institutions (J.T.S. unpublished) as well being reported to individuals in a randomised controlled trial to determine if the knowledge of the presence and extent of AAC may lead to modification of diet and lifestyle behaviours.^22^ Part of the reason there has been little clinical update has been uncertainty over the prognostic importance of AAC, this has recently been demonstrated for CVD events and all-cause mortality in the general population and those with kidney disease.^11^ Furthermore, AAC has recently been shown to be associated with higher risk of non-cardiovascular events such as fracture and late-life dementia hospitalisations and deaths.^23^^,^^24^ A major reason limiting the exploration of the clinical utility of AAC from these images has been the lack of automated assessment.

In the real-world assessment, ML-AAC-24 scoring was able to directly link ML-AAC groups with subsequent cardiovascular outcomes, confirming that the approach identifies large proportions of people at substantially higher cardiovascular risk and poorer long-term prognosis. Higher ML-AAC-24 as predicted by the algorithm were strongly and robustly associated with the primary composite outcome of MACE, as well as all of the individual components of all-cause mortality, hospitalised myocardial infarction or cerebrovascular disease, in addition to a range of secondary cardiovascular diagnoses. These associations remained strong when adjusted for multiple baseline covariates, even after excluding individuals with prior cardiovascular diagnoses. These data demonstrate that the ML approach can accurately classify a patient's extent of AAC, and hence CVD risk when undertaking DXA-based lateral spine imaging currently collected to detect spine fracture.

Increasingly, clinical guidelines are recommending performing this vertebral fracture assessment (VFA) imaging at the time of DXA testing to identify asymptomatic vertebral fractures. As such automated assessment of the presence and extent of AAC with similar accuracies to imaging specialists provides the possibility of large-scale screening for asymptomatic CVD. The observed hazard ratios for increased ML-AAC-24 align closely with those that were reported in a recent meta-analysis of 46 cohorts of 36,092 participants.^11^ In 5 studies (n = 6754) reporting three or more groups of AAC compared to no/low AAC groups, the moderate AAC groups had a pooled RR of 1.40 (1.06–1.84) and 2.06 (1.48–2.88) for high AAC groups. This is very similar to our moderate ML-AAC-24 adjusted HR of 1.54 (1.30–1.83) and high ML-AAC-24 of 2.11 (1.77–2.51). Importantly we show that AAC scores estimated using an automated approach in a real-world clinical setting robustly predict incident CVD outcomes, which makes widespread application of AAC assessment in clinical practice more feasible.

As per Koo and Li's guidelines,^25^ the intraclass correlation ICC between the ground truth scores and ML-AAC-24 for the different test sets ranged from 0.76 to 0.90 and can be categorised as good agreement for the four makes of bone density machines in clinical use over the last 30 years. When comparing the three AAC group analysis for the GE Lunar Prodigy and iDXA images, our Cohen's weighted kappa between the ML-AAC vs. imaging specialists was 0.687 (95% CI 0.665–0.709) which was very similar to the Cohen's weighted kappa of 0.682 (95% CI 0.597–0.766) for the three AAC groups between two readers trained by J.T.S. in a subset of images annotated by both readers (n = 175).

We chose to train the algorithm on the largest dataset from a single machine (Hologic 4500A) read by the most experienced expert (J.T.S) and tested on other bone density machine makes and models. This approach was selected given fine-tuning a model which has already learned to map images to AAC scores is more plausible than training from scratch, especially when the size of the datasets with expert assessment are limited. In future if larger datasets of several thousand images with expert assessment are available, training within specific machine makes and models (for example, Hologic Horizon vs. Discovery) may be possible.

The agreement and accuracies across the different makes and models of bone densitometers were similar to Reid et al.^19^ investigating training and testing on GE machines in a smaller dataset of the same scans used in the current paper. However, for the GE Lunar Prodigy images the ICC (0.78) for the 817 images used in our study was at the lower ranges of the previously reported 95% confidence intervals of the ICC of 0.78–0.91. This may be due to the training on Hologic images, differences in the ML approaches or differences in the images/size of datasets used. Generally, there was better agreement and accuracies on the images from the newer scanners and these differences may be attributed to the superior resolution of these scanners.

However, whilst these results are promising, there remain many areas for improvements. For example, the ML tended to underestimate the AAC-24 scores compared to human assessment, leading to greater misclassification into lower AAC groups, suggesting further development is needed. This underestimation may lead to weaker associations with clinical outcomes but will need further investigation in larger datasets with clinical outcomes. When looking at the activation maps of the outliers (>2.5 SD from the mean difference between AAC-24 and ML-AAC-24) we identified a number of cases where the ML-AAC-24 scores failed to identify and score iliac artery calcifications, when these were adjacent to the L4 region. We continue to investigate other potential causes of discrepancies such bowel gas overlying the aorta and the ribs being mistaken for AAC adjacent to the L1 region in people with hyperlordosis. Similarly, we are investigating whether vertebral fractures, scoliosis, osteophytes and rotation leading to the aorta being partly covered by the spine, may be affecting the agreement between AAC-24 and ML-AAC-24 scores. We are also increasing the size of the datasets and adding more granular manually assessed calcification data, such as the adjacent lumbar region where calcifications were observed, and whether these were in the anterior or proximal aortic wall to improve the accuracies of the algorithm. Finally, whilst the algorithm generally had a high level of sensitivity, specificity and accuracy identifying people with high AAC, the performance for identifying individuals with less extensive AAC was lower. This suggests further work is needed to improve these measures.

In this work, we used a state-of-the-art convolutional neural network model *EfficientNet*^20^ for feature extraction. EfficientNets have not only achieved superior classification accuracy on ImageNet compared to the other commonly used networks such as DenseNet,^26^ InceptionNet^27^ and ResNet,^28^ but also have significantly fewer training parameters. Moreover, EfficientNets are found to transfer well to other datasets.^20^ The EfficientNet-based model achieved promising results and validate the reliability and utility of the models across different DXA manufacturers and models even in the case of low-quality Lunar Prodigy scans. We also used 10-fold stratified cross-validation technique that takes into account the distribution of the target variable. It ensures that each subset of the data has a similar distribution of the target variable, which can help in improving the accuracy of a model's prediction.

### Limitations

We only had access to manually assessed VFA images from the two largest manufactures and the algorithm may not perform as well on other manufacturers' VFA images. However, Hologic and GE account for the vast majority of DXA machine sales. Additionally, we did not have access to real-world examples with long-term clinical follow up for Hologic bone density machines similar to what was available for the Manitoba dataset. However, we did have the long-term outcomes for the Perth Longitudinal Study of Ageing Women where ML-AAC was similarly associated with mortality outcomes to human assessment and predicted mortality outcomes in images that had been captured 5 years after the last human assessments of AAC. Also, we did not look at how patient characteristics such as age, ethnicity, sex and BMI may affect performance. However, the associations between ML-AAC-24 groups and MACE outcomes were independent of these characteristics. Secondly, the Manitoba registry lateral spine images were captured based on age and an increased a priori risk of osteoporosis and fracture and thus may not represent younger individuals and those with better bone structure. Additionally, as we used a MACE composite outcome that includes all-cause mortality this may weaken the association between ML-AAC-24 and the outcome due to non-cardiovascular causes of death. However, despite this we identified strong and robust associations between ML-AAC-24 and the MACE composite outcome as well as all of the individual cardiovascular outcomes.

### Strengths

We had access to the largest manually assessed set of VFA images from widely-used bone density machine models in clinical practice over the last three decades. Importantly, these images were read by a globally-recognised expert in assessing AAC-24 (JTS) or people trained by J.T.S. providing reliable and consistent scoring for ML. Further strengths include i) the use of advanced and efficient ML models to quantify AAC, and, ii) the generation of visual explanations (in the form of localisation heatmaps) which add to the explainability of the model. We believe that clinical trials comparing patient and physician behaviour, treatment and outcomes based on ML-AAC-24 reporting are now warranted.

In conclusion, we found substantial agreement between trained imaging expert and machine-learning AAC-24 scores irrespective of the make and model of DXA machine. Since these images and automated scores can be rapidly and easily acquired at the time of bone density testing this may lead to novel approaches for early cardiovascular disease detection and disease monitoring in routine clinical practice settings.

## Contributors

SZG, DS, WDL, JTS, JRL conceived and designed the study; DS, KZ, RLP, DPK, JTS, JRL acquired funding; RLP, KZ, SR, WHL, JRL, JTS, WDL collected the data; NS, MS, SZG, WDL, JRL analysed the data; NS, SZG, DS, BAM, MJJ, WDL, JTS, JRL prepared the manuscript with input from all authors; ZG and JRL verified the underlying data and NS, SZG, WDL, JTS, MS, JRL had the primary responsibility for the final content. All authors provided intellectual input and edited the paper. All authors read and approved the final manuscript.

## Data sharing statement

The data that support the findings of this study are available from the relevant Data Custodians, including the Manitoba Population Research Data Repositor, Department of Health Human Research Ethics Committee, and the Department of Health Research Governance Office. Restrictions apply to the availability of these data, which were used under license for the current study, and so are not publicly available. However, data will be made available from the authors upon reasonable request once permission of the relevant Data Custodians have been obtained.

## Declaration of interests

DPK has received a grant from Solarea Bio and royalties from Wolters Kluwer. DPK sits on the Scientific Advisory Boards of Solarea Bio, Pfizer and Reneo and has participated on the Data Safety Monitoring Board for the AgNovos Healthcare osteoporosis treatment trial. All other authors have no disclosures to declare.
