# Supplementary material for: Machine learning for abdominal aortic calcification assessment from bone density machine-derived lateral spine images
Source: eBioMedicine. 2023 Jul 11;94:104676. doi: 10.1016/j.ebiom.2023.104676 (PMC10435763; doi:10.1016/j.ebiom.2023.104676)
Supplement: Supplementary Material [file mmc1.docx]

**Supplementary Text**

**Supplementary Section 1. Details of the datasets used**

**Hologic 4500A dataset (n=1,914).** The Hologic 4500A dataset comprised of 1,914 VFA scans captured from the Calcium Intake Fracture Outcome Study^(1)^ that was followed up for an additional 10 years as part of the Perth Longitudinal Study of Ageing Women (PLSAW). Imaging specialist (J.T.S. ^(2-4)^) assessed AAC-24 from lateral spine images were obtained in 1998/1999 and 2003/2004. A further 582 images without AAC-24 assessment were available in 2008.

**Hologic Horizon dataset (n=508).** There were two Hologic Horizon datasets, the first dataset comprised 326 images acquired among patients (mean age 71 years, 94.6% women) referred for bone densitometry at Park Nicollet Clinic of HealthPartners Inc, Minneapolis, MN between January 1, 2017 and March 31, 2020; the second dataset of 182 images is part of a study in ambulatory community-dwelling Australian men and women, aged 60–80 years, recruited from the general population in metropolitan Perth and surrounding areas in Western Australia performed at Gairdner Bone Densitometry Services, Sir Charles Gairdner Hospital. All images were read by a single experienced investigator J.T.S ^(2-4)^ blinded to outcomes.

**GE datasets (n=817 for Lunar Prodigy and n=1,773 for iDXA).** The GE datasets were from the Manitoba Bone Density Program that created a database of 12,742 de-identified images of patients who qualified for vertebral fracture assessment as part of osteoporosis assessment during 2010-2017. The images in this registry were acquired through GE Lunar Prodigy and iDXA machines in the province of Manitoba, Canada. Further details regarding the baseline characteristics of the study group can be found in the work published by Reid et al^(5)^. For the current analysis, we used 2,590 selected images with human assessed AAC14 scores, with **817** (31.5%) acquired on **Lunar Prodigy** and the rest **1,773** (68.5%) on **iDXA machines**. Details of the labelled images for AAC-24 scores are provided in **Supplementary Table 1**.

**Supplementary section 2. Additional methodology details for the automated AAC-24 methodology**

*Image pre-processing*

The VFA scans used in this work were pre-processed before being input into the machine learning model for training/validation/testing. Only single energy (SE) scans were available for the *Hologic* machines, whereas for *GE* machines both SE and dual energy (DE) scans were available. For scans from Hologic machines, the top 50% portion of all the scans was cropped to extract the region of interest i.e., lower lumbar region. For GE scans, we followed the approach of Reid et al^(5)^ to also crop 40% of the left side and 10% of the right of each image. The cropped images were resized to 300 x 300 pixels using the nearest neighbor interpolation strategy. Finally, the image pixels were rescaled between 0 and 1. To increase the size of our training dataset, we augmented our data by applying affine transformations on the images, such as scaling [+20%, -20%], translation [+20%, -20%], rotation [+15°, -15°] and shear [0.01°, 0.05°]. We used *TorchVision Transforms* library to apply a set of random affine transforms to each batch of images during model training.

*10-Fold cross-validation and testing*

To effectively analyse the reliability and generalisability of our approach we first trained our model on the SE VFA scans from the Hologic-4500A dataset (n=1,914) and then independently tested it on the SE images from one Hologic Horizon dataset (n=339), and a second Hologic Horizon dataset (n=182). We performed 10-fold stratified cross-validation^(6, 7)^ on the Hologic-4500A dataset, which involved splitting the sets of images into 10 random sets (folds) such that each set maintains the same distribution of AAC scores. Each time the model was trained on 9 folds (n=1,722) and tested on the remaining one (n=172). This process was repeated 10 times and then the performance of the model on all test scans reported (n=1,914). We then tested the models trained on the Hologic-4500A dataset on the held-out Hologic Horizon datasets. The images in our test sets not only had different resolutions compared to the training images but also had different distributions of AAC scores (as shown in Table-1).

There are visible differences between the VFA scans obtained from Hologic and GE machines. In particular, the scans from GE have a GE SmartScan feature limiting the field of view to reduce radiation dose that appears as a black mask on the regions surrounding the spine and aorta. This black masking is not part of VFA scans obtained from *Hologic* machines and the images which we used to train our model. Therefore, to help our model adapt to GE scans, we fine-tuned it using a combination of 2,331 iDXA and Lunar Prodigy scans from the Manitoba bone density registry. Similar to our training and test settings for *Hologic-4500A, we performed 10-fold stratified cross-validation,* which involved fine-tuning our model on 2,331 (90%) training examples and testing on 259 (10%) scans, 10 times. Importantly, we fine-tuned and tested our models independently for the SE and DE scan modes.

*Model and training parameters*

Various hyperparameters including, learning rate, batch size, number of neurons in the fully connected layers of our regression network, were chosen through empirical analysis. We optimised our networks for Mean Square Error (MSE), using the Adam optimiser^(8)^, with a learning rate of 5 x 10^-4^. We used a batch size of 20 to train our model on SE images from Hologic-4500A. We fine-tuned our models on SE and DE images from GE, maintaining the same batch size (i.e., 20) but a lower learning rate (1 x 10^-4^). We performed early stopping when the validation loss stopped decreasing and then assessed our model on the test set. For all experiments, we used the PyTorch Machine Learning Library^(9)^.

*Activation maps for the models*

Finally, using Grad-CAM^(10)^ we analysed the activation maps of our model, which reflect the regions of image where the model paid attention to make a decision/generate a score. These activation/heat maps can be useful for understanding/explaining the rationale behind the model generated score.

*Using DE/SE scans from GE Machines*

In general, combining different types of images also poses challenges in terms of pre-processing and model training. In essence, DE images are superior to SE images in terms of the information they carry. That is perhaps why, adding SE images only adds to the ‘redundancy’ not the ‘relevance’ of information.(11) We noticed in our experiments that combining SE and DE images enables the network to generalize slightly better on both types of images, but it comes at the cost of reduced performance on either SE or DE modality. e.g. in our experiments combining SE and DE images the Pearson correlation on GE iDXA DE test images was 0.89, which is lower than what we have on our test set of 0.91 after training on GE only.

**Supplementary text section 3. Covariates in the Manitoba registry**

Multiple covariates that could contribute to cardiovascular events were identified and included in the adjusted models. This included age, sex, body mass index (BMI), current tobacco smoking, high alcohol intake (3 or more drinks per day) assessed at the time of VFA; diagnoses of diabetes and hypertension in the last 3 years (linked physician claims and hospitalisation records); prior myocardial infarction or cerebrovascular disease at any time since 1984 (linked hospitalisation records). Social determinants related to income (lower two quintiles versus upper three quintiles), area of residence (rural versus urban), and ethnicity (self-reported white versus non-white) were also included(12, 13). Medications used for at least 6 months in the year prior to the index date were identified from the province-wide retail pharmacy database (glucocorticoid, statin, nonselective beta blocker, selective beta blocker, angiotensin receptor blocker, ACE inhibiter, aldosterone blocker, loop diuretic, thiazide diuretic, digoxin, calcium channel blocker, long-acting nitrate and oral vitamin K anticoagulant), (14, 15).

**Supplementary Table 1.** Details on the machines and numbers of imaging specialists labelled scans available.

| **Stage** | **Training**  **& testing** | **Testing**  **only** |  | **Fine tuning**  **& testing** | **Fine tuning**  **& testing** | **Clinical assessment only** |
| --- | --- | --- | --- | --- | --- | --- |
| **Bone Density Machine** | **Hologic** | |  | **GE** | | **GE** |
| **Model** | **4500A** | **Horizon** | **Lunar Prodigy** | | **iDXA** | **Prodigy & iDXA** |
| **Single-energy VFA’s** | 1,914*† | 508 | 817 | | 1,773 | 8,525 |
| **Dual-energy VFA’s** | N/A | N/A | 817 | | 1,773 | 8,525 |
| **Low AAC (AAC-24 < 2)** | 764 (39.9%) | 293 (57.7%) | 397 (48.6%) | | 756 (42.6%) | No labelled scans |
| **Moderate AAC (AAC-24 2-5)** | 714 (37.3%) | 165 (32.5%) | 181 (22.2%) | | 408 (23.0%) | No labelled scans |
| **High AAC (AAC-24 ≥ 6)** | 436 (22.8%) | 50 (9.8%) | 239 (29.2%) | | 609 (34.4%) | No labelled scans |
| Abbreviations; AAC-24 Abdominal Aortic Calcification 24-point scores, GE General Electric, VFA vertebral fracture assessment images.  * 1082 VFA captured in 1998/1999 were used for comparison to human AAC-24 assessment for 14.5 year mortality outcomes.  † 582 VFA captured in 2008 with no labelled scans AAC-24 assessment for 5-year mortality outcomes. | | | | | | |

**Supplementary Table 2.** Characteristics of the Manitoba registry-based cohort stratified by automated abdominal aortic calcification-24 groups via machine learning (ML-AAC-24).

| **Characteristics** | **Low** | **Moderate** | **High** |
| --- | --- | --- | --- |
| Number (%) | 3400 (39.7) | N=2840 (33.2) | N=2325 (27.1) |
| **Age (years)** | 73.8 ± 6.4 | 76.3 ± 6.5 | 78.3 ± 6.5 |
| **Sex (female)** | 3242 (95.4) | 2642 (93.0) | 2168 (93.2) |
| **Body mass index (kg/m^2^)** | 26.2 ± 5.1 | 26.6 ± 5.0 | 25.8 ± 4.7 |
| **Smoking** | 134 (3.9) | 227 (8.0) | 330 (14.2) |
| **High alcohol intake** | S (<1) | 8 (0.2) | 7 (0.3) |
| **Lower income** | 997 (29.3) | 915 (32.2) | 879 (37.8) |
| **Rural residency** | 824 (24.2) | 788 (27.8) | 653 (28.1) |
| **Non-white ethnicity** | 173 (5.1) | 111 (3.9) | 69 (3.0) |
| **Comorbidities** |  |  |  |
| **Diabetes †** | 316 (9.3) | 390 (13.7) | 405 (17.4) |
| **Hypertension diagnosis †** | 1497 (44.0) | 1626 (57.3) | 1555 (66.9) |
| **Prior MI or cerebrovascular disease ‡** | 107 (3.1) | 204 (7.2) | 295 (12.7) |
| **Medication use in the prior year** |  |  |  |
| **Glucocorticoid** | 187 (5.5) | 181 (6.4) | 134 (5.8) |
| **Statin** | 768 (22.6) | 907 (31.9) | 995 (42.8) |
| **Nonselective beta blocker** | 40 (1.2) | 54 (1.9) | 54 (2.3) |
| **Selective beta blocker** | 308 (9.1) | 412 (14.5) | 529 (22.8) |
| **Angiotensin receptor blocker** | 460 (13.5) | 534 (18.8) | 503 (21.6) |
| **ACE inhibitor** | 444 (13.1) | 526 (18.5) | 520 (22.4) |
| **Aldosterone blocker** | 19 (0.6) | 21 (0.7) | 46 (2.0) |
| **Loop diuretic** | 105 (3.1) | 173 (6.1) | 199 (8.6) |
| **Thiazide diuretic** | 418 (12.2) | 441 (15.5) | 424 (18.2) |
| **Digoxin** | 24 (0.7) | 35 (1.2) | 44 (1.9) |
| **Calcium channel blocker** | 467 (13.7) | 559 (19.7) | 640 (27.5) |
| **Long acting nitrate** | 18 (0.5) | 45 (1.6) | 65 (2.8) |
| **Oral anticoagulant se** | 83 (2.4) | 119 (3.9) | 122 (5.2) |

Data expressed as mean ± SD or n(%). S, suppressed small cell size (<6).

* ML-AAC-24 groups, low <2, moderate 2-5, high >6.

† Diagnosis codes over the last 3 years

‡ Diagnosis codes since 1984

**Supplementary Table 3.** Diagnosis codes International Classification of Diseases, Ninth Revision, Clinical Modification (ICD9-CM) and International Classification of Diseases, Tenth Revision, Canadian Enhancements (ICD10-CA) diagnosis codes; Procedure codes ICD-9 and Canadian Classification of Health Interventions (CCI).

| Primary outcomes |
| --- |
| myocardial infarction; ICD-9-CM 410, ICD-10-CA I21  cerebrovascular disease: ICD-9-CM 433-435, ICD-10-CA I63-66, G45 |
|  |
| Secondary outcomes |
| coronary heart disease: ICD-9-CM 410-414, ICD-10-CA I20-I25  heart failure: ICD-9-CM 428, ICD-10-CA I50  peripheral arterial disease: ICD-9-CM 440-443, ICD-10-CA I70-73  coronary bypass grafting: ICD-9 36.10, 36.11, 36.12, 36.13, 36.14, 36.19, CCI 1.IJ.76.^^  coronary angioplasty or coronary stent ICD-9 36.01, 36.02, 36.05, 36.06, CCI 1.IJ.50.^^, 1.IJ.57.^^ |

**Supplementary Table 4.** Performance of the predicted abdominal aortic calcification-24 scores (ML-AAC-24) to classify dichotomous groups based on severity.

| **Performance measures** | **Low vs. moderate-high AAC** | **Low-moderate vs. high AAC** |
| --- | --- | --- |
| **Accuracy (%, 95% CI)** | 81.0 (79.8 to 82.0) | 87.9 (87.0 to 88.8) |
| **Sensitivity (%, 95% CI)** | 82.9 (81.5 to 84.3) | 65.7 (63.1 to 68.2) |
| **Specificity (%, 95% CI)** | 78.5 (76.7 to 80.2) | 96.0 (95.3 to 96.6) |
| **PPV (%, 95% CI)** | 82.9 (81.7 to 84.0) | 85.6 (83.4 to 87.4) |
| **NPV (%, 95% CI)** | 78.5 (77.0 to 79.9) | 88.5 (87.7 to 89.3) |

ML-AAC-24: Low= 0 or 1, moderate= 2-5, and high= ≥6. PPV Positive predictive value, NPV Negative predictive value.

**Supplementary Table 5.** Quantitative results from 10-fold cross-validation on labelled Lunar Prodigy and iDXA single-energy test scans.

| GE Lunar Prodigy | Low AAC  (n=397) | Moderate AAC (n=181) | High AAC  (n=239) | Average |
| --- | --- | --- | --- | --- |
| **Accuracy (%)** | 76.4 | 68.2 | 84.9 | 76.5 |
| **Sensitivity (%)** | 74.8 | 43.6 | 64.0 | 60.8 |
| **Specificity (%)** | 77.9 | 75.2 | 93.6 | 82.2 |
| **Negative Predictive Value (%)** | 76.6 | 82.4 | 86.3 | 81.8 |
| **Positive Predictive Value (%)** | 76.2 | 33.3 | 80.5 | 63.3 |
| **GE iDXA** | **Low AAC**  **(n=756)** | **Moderate AAC (n=408)** | **High AAC**  **(n=609)** | **Average** |
| **Accuracy (%)** | 85.7 | 77.6 | 89.8 | 84.4 |
| **Sensitivity (%)** | 86.2 | 54.4 | 79.5 | 73.4 |
| **Specificity (%)** | 85.3 | 84.5 | 95.3 | 88.4 |
| **Negative Predictive Value (%)** | 89.3 | 86.1 | 89.9 | 88.4 |
| **Positive Predictive Value (%)** | 81.4 | 51.3 | 89.8 | 74.2 |

Abbreviations; AAC Abdominal Aortic Calcification, AAC-24 Abdominal Aortic Calcification 24-point scores GE General Electric, SE Single-Energy.


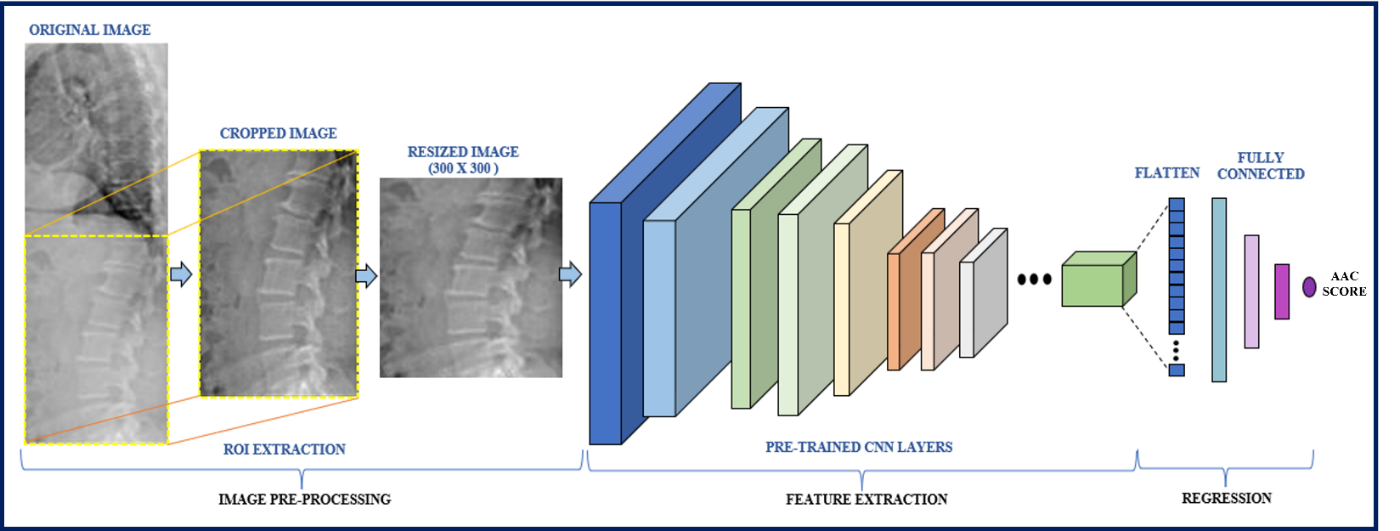


**Supplementary Figure 1.** Overview of the framework to automatically quantify Abdominal Aortic Calcification 24 scores from DXA images.

**
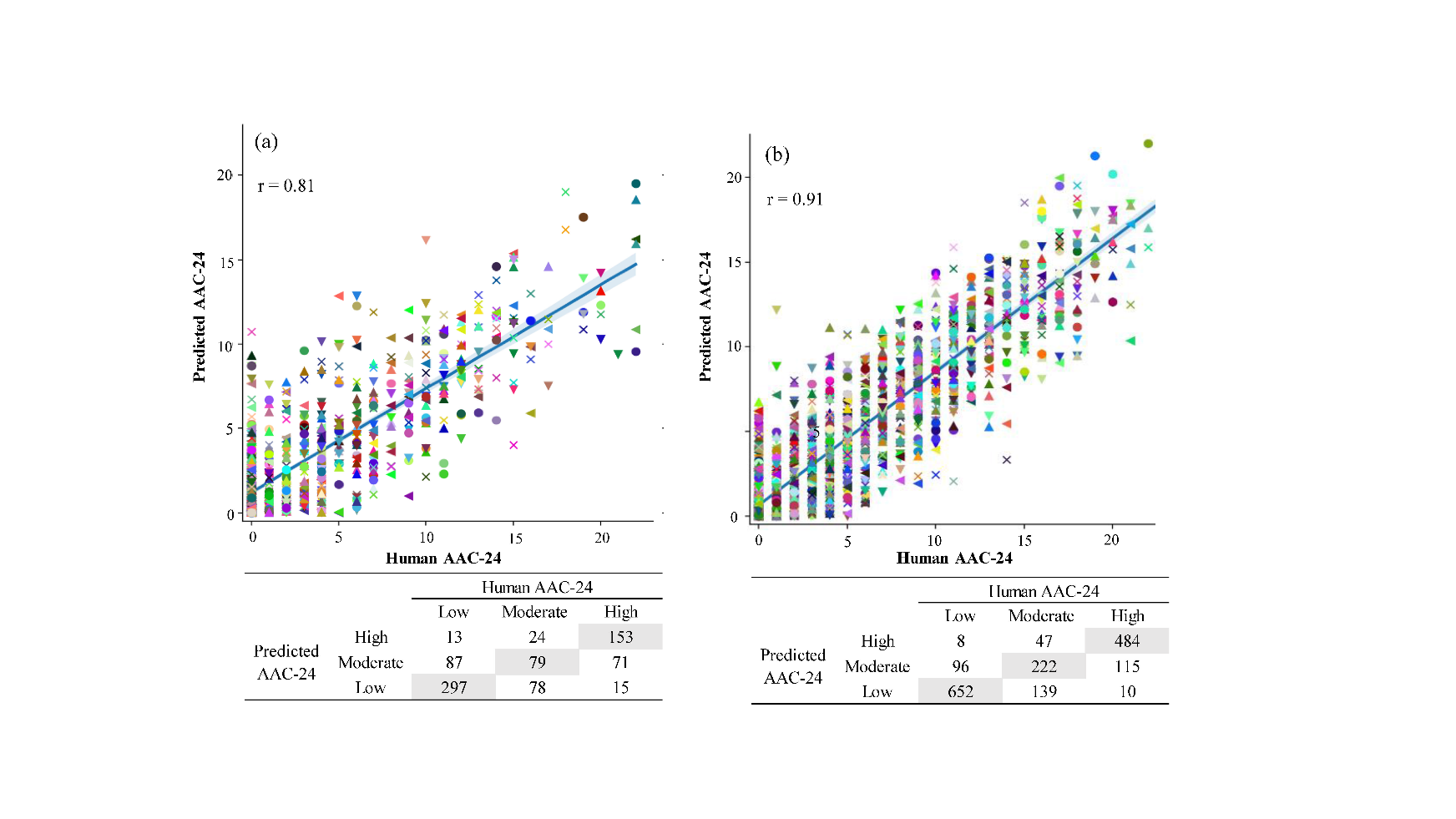
**

**Supplementary Figure 2.** Scatter plots of imaging specialist assessments of AAC-24 scores versus the ML computed AAC-24 scores (a) GE Lunar Prodigy singleenergy, and (b) GE iDXA single-energy. The blue line represents the regression line and ‘r’ is the Pearson correlation coefficient. Below each plot is the confusion matrix for three-class classification of the AAC scores.


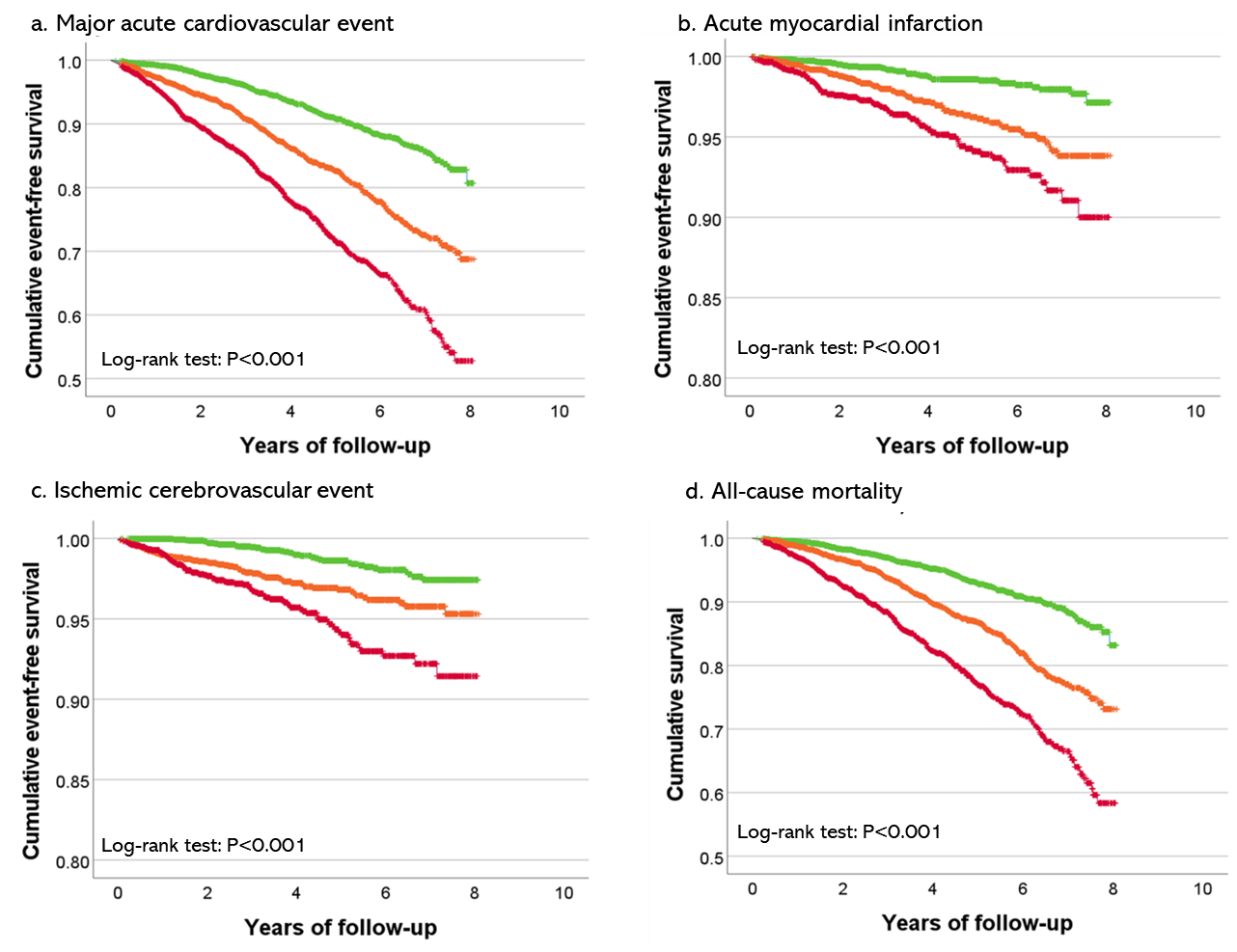


**Supplementary Figure 3.** Kaplan-Meier curves for event-free survival proportions from machine learning computed AAC groups categorised as low (green), moderate (orange), or high (red). Vertical lines represent censored observations in the Manitoba registry-based cohort.

**References**

1. Prince RL, Devine A, Dhaliwal SS, Dick IM. Effects of calcium supplementation on clinical fracture and bone structure: results of a 5-year, double-blind, placebo-controlled trial in elderly women. Archives of internal medicine. 2006;166(8):869-75.

2. Schousboe JT, Taylor BC, Kiel DP, Ensrud KE, Wilson KE, McCloskey EV. Abdominal aortic calcification detected on lateral spine images from a bone densitometer predicts incident myocardial infarction or stroke in older women. Journal of Bone and Mineral Research. 2008;23(3):409-16.

3. Schousboe JT, Wilson KE, Kiel DP. Detection of abdominal aortic calcification with lateral spine imaging using DXA. Journal of Clinical Densitometry. 2006;9(3):302-8.

4. Schousboe JT, Wilson KE, Hangartner TN. Detection of aortic calcification during vertebral fracture assessment (VFA) compared to digital radiography. PloS one. 2007;2(8):e715.

5. Reid S, Schousboe JT, Kimelman D, Monchka BA, Jozani MJ, Leslie WD. Machine learning for automated abdominal aortic calcification scoring of DXA vertebral fracture assessment images: A pilot study. Bone. 2021;148:115943.

6. Zeng X, Martinez TR. Distribution-balanced stratified cross-validation for accuracy estimation. Journal of Experimental & Theoretical Artificial Intelligence. 2000;12(1):1-12.

7. Kohavi R, editor A study of cross-validation and bootstrap for accuracy estimation and model selection. Ijcai; 1995: Montreal, Canada.

8. Kingma DP, Ba J. Adam: A method for stochastic optimization. arXiv preprint arXiv:14126980. 2014.

9. Paszke A, Gross S, Massa F, Lerer A, Bradbury J, Chanan G, et al. Pytorch: An imperative style, high-performance deep learning library. Advances in neural information processing systems. 2019;32.

10. Selvaraju RR, Cogswell M, Das A, Vedantam R, Parikh D, Batra D, editors. Grad-cam: Visual explanations from deep networks via gradient-based localization. Proceedings of the IEEE international conference on computer vision; 2017.

11. Peng H, Long F, Ding C. Feature selection based on mutual information criteria of max-dependency, max-relevance, and min-redundancy. IEEE Transactions on pattern analysis and machine intelligence. 2005;27(8):1226-38.

12. Manitoba Centre for Health Policy. Concept: Urban / Rural Definitions.2002. Available from: <http://mchp-appserv.cpe.umanitoba.ca/viewConcept.php?conceptID=1253> (Last accessed September 19, 2021).

13. Manitoba Centre for Health Policy. Income quintiles.2003. Available from: <http://mchp-appserv.cpe.umanitoba.ca/viewDefinition.php?definitionID=102882> (Last accessed February 10, 2019).

14. Kozyrskyj AL, Mustard CA. Validation of an electronic, population-based prescription database. Ann Pharmacother. 1998;32(11):1152-7.

15. Metge C, Black C, Peterson S, Kozyrskyj AL. The population's use of pharmaceuticals. Med Care. 1999;37(6 Suppl):JS42-JS59.
